# Supplementary material for: Buccal Swab Samples from Japanese Brown Cattle Fed with Limonite Reveal Altered Rumen Microbiome
Source: Animals (Basel). 2024 Jul 3;14(13):1968. doi: 10.3390/ani14131968 (PMC11240510; doi:10.3390/ani14131968)
Supplement: Supplementary file 1 [file animals-14-01968-s001.zip › Harakawa et al Figure_S.pptx]

## Slide 1
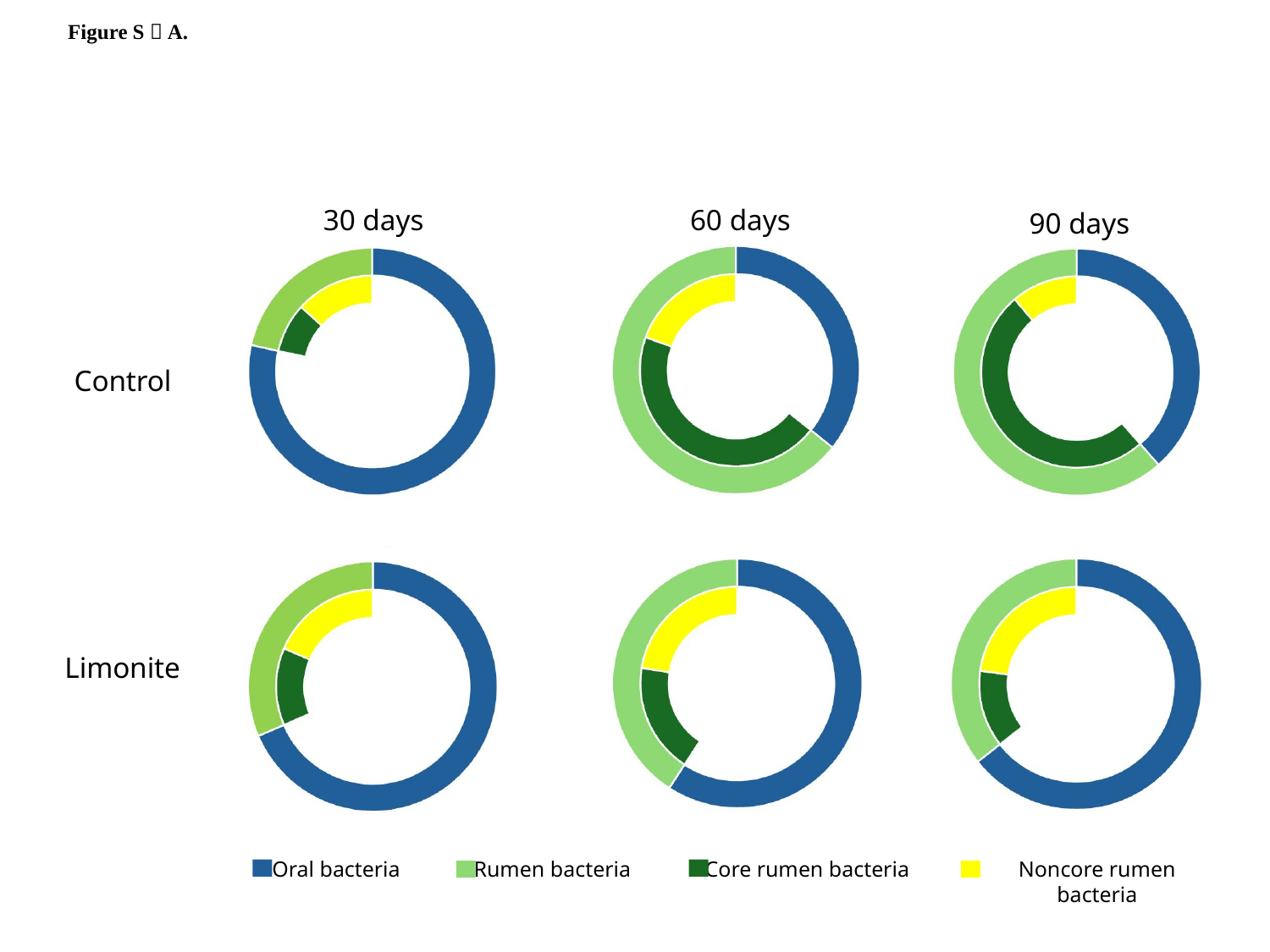

Figure S１A.
30 days
60 days
90 days
Control
Limonite
Rumen bacteria
Oral bacteria
Core rumen bacteria
Noncore rumen bacteria

## Slide 2
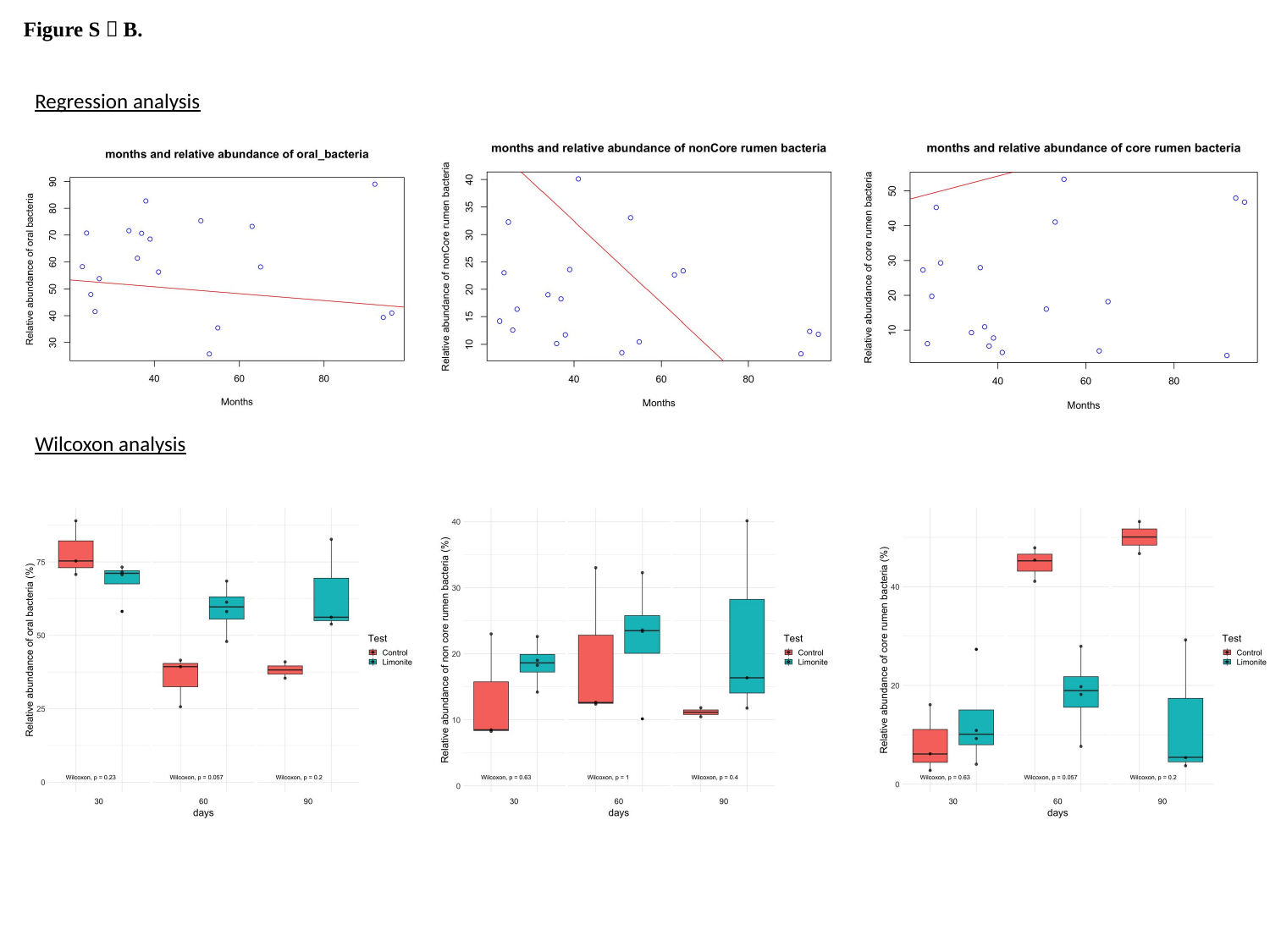

Figure S１B.
Regression analysis
Wilcoxon analysis

## Slide 3
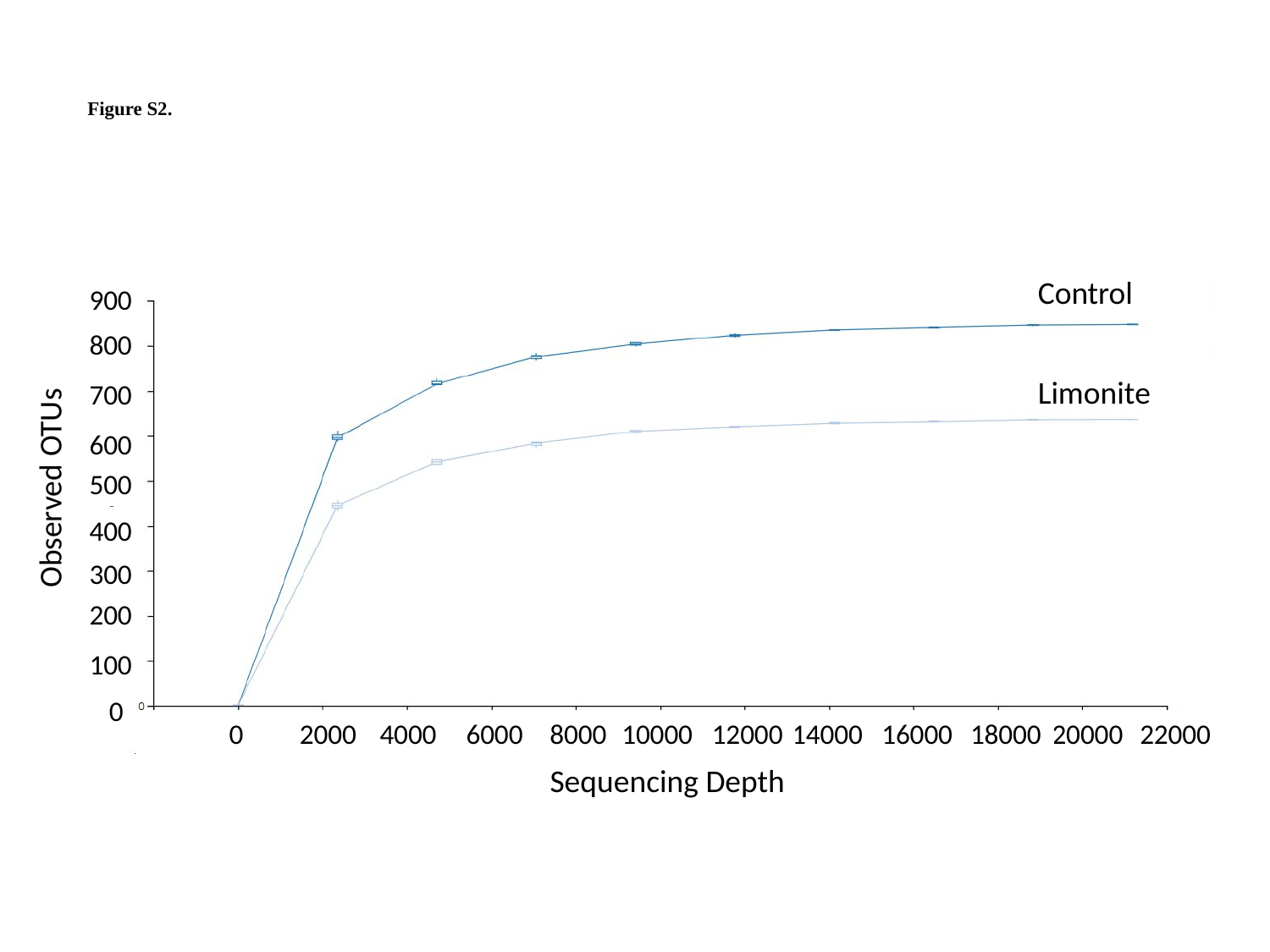

Figure S2.
Control
900
800
Limonite
700
600
500
Observed OTUs
400
300
200
100
0
0
2000
4000
6000
8000
10000
12000
14000
16000
18000
20000
22000
Sequencing Depth
